# Lemmel’s Syndrome: Usual Presentation of an Unusual Diagnosis

**DOI:** 10.7759/cureus.7698

**Published:** 2020-04-16

**Authors:** Michelle Bernshteyn, Suman Rao, Anuj Sharma, Umair Masood, Divey Manocha

**Affiliations:** 1 Internal Medicine, State University of New York Upstate Medical University, Syracuse, USA; 2 Gastroenterology, State University of New York Upstate Medical University, Syracuse, USA

**Keywords:** endoscopy, diverticulum, lemmel

## Abstract

Lemmel’s syndrome causes obstructive jaundice in the absence of stones or tumors. The most common cause is the presence of periampullary diverticula which arise within 2-3 cm from the ampulla of Vater. Diverticula may be extramural or intramural. Despite current practice of obtaining imaging studies such as ultrasound, CT, and MRI, endoscopic retrograde cholangiopancreatography (ERCP) is the gold standard diagnostic test. Lemmel’s syndrome should be considered when pancreaticobiliary disease is suspected. We present a case in which our patient presented with abdominal pain, fever, and transaminitis who underwent ERCP which was successful in diagnosis of Lemmel’s syndrome and its treatment. Although rare, it is imperative for physicians to recognize this syndrome in order to deliver prompt care.

## Introduction

Although it is a reflex to associate the pathophysiology of obstructive jaundice to choledocholithiasis and its counterparts, there exists a realm of pathophysiology centered around periampullary diverticula (PAD) which presents with similar signs and symptoms. Lemmel’s syndrome constitutes the presence of obstructive jaundice in the absence of choledocholithiasis or pancreaticobiliary tumors [1]. PAD is the most common cause of Lemmel’s syndrome [2]. The incidence of a PAD is 1%-27% [1]. They usually result in malfunctioning of the sphincter of Oddi, or mechanically obstruct outflow through the common bile duct [3]. Clinical symptoms consist of right upper quadrant pain, and laboratory workup would reveal elevated bilirubin levels, elevated liver enzymes, and/or pancreatic enzymes depending on involvement of the ampulla of Vater [4]. Currently, a side-viewing endoscopic retrograde cholangiopancreatography (ERCP) is recommended in diagnosing PAD leading to Lemmel’s syndrome [1]. In symptomatic patients, endoscopic extraction and surgery, such as a diverticulectomy, are usually modalities of treatment [5].

## Case presentation

A 57-year-old male with a past medical history significant for type II diabetes mellitus, gastroesophageal reflux disease, psoriasis, and laparoscopic cholecystectomy complicated by liver abscess and Escherichia coli bacteremia one year prior presented to the hospital due to five days of right upper quadrant pain, fever, and elevated liver function tests which was discovered by his primary care provider.

**Figure 1:**
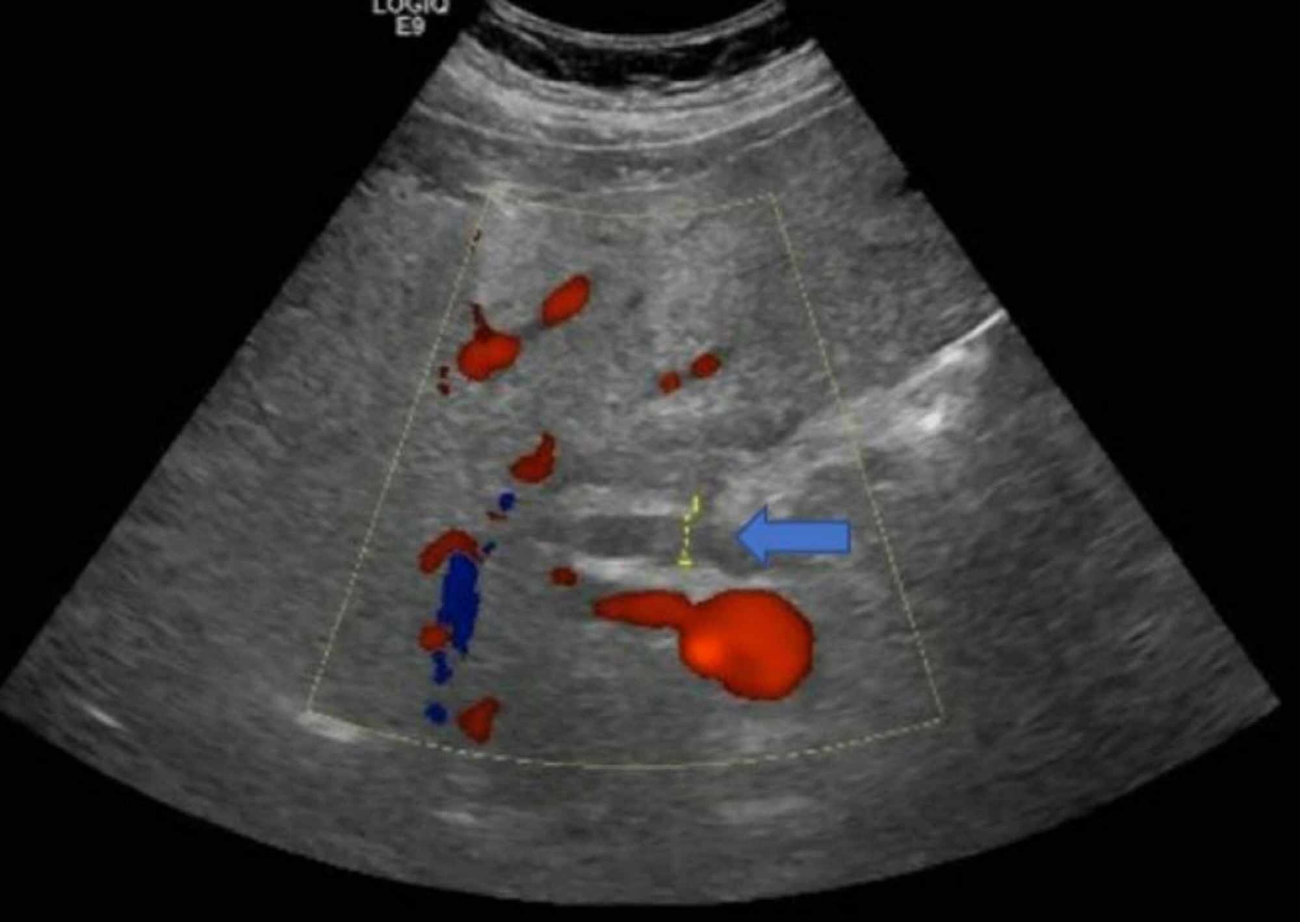
Common bile duct measuring approximately 1.2 cm.

In the emergency department, the patient was found to have a temperature of 38°C and was tachycardic at 114 beats per minute. Laboratory results demonstrated elevated alkaline phosphatase of 194 IU/L (normal: 40-129 IU/L), alanine aminotransferase of 106 U/L (normal <41 U/L), aspartate aminotransferase of 260 U/L (normal <40 U/L), and total bilirubin of 5.5 mg/dL (normal <1.2 mg/dL). Ultrasound of the abdomen demonstrated multiple hypoechoic lesions in the right lobe of the liver. There was a 6.9 x 2.3 x 2.5 cm heterogeneous area with multiple hyperechoic foci projecting in the region of the gallbladder fossa. There was also evidence of common bile duct dilation to 1.2 cm (normal <0.7 cm) (Figure 1).

**Figure 2:**
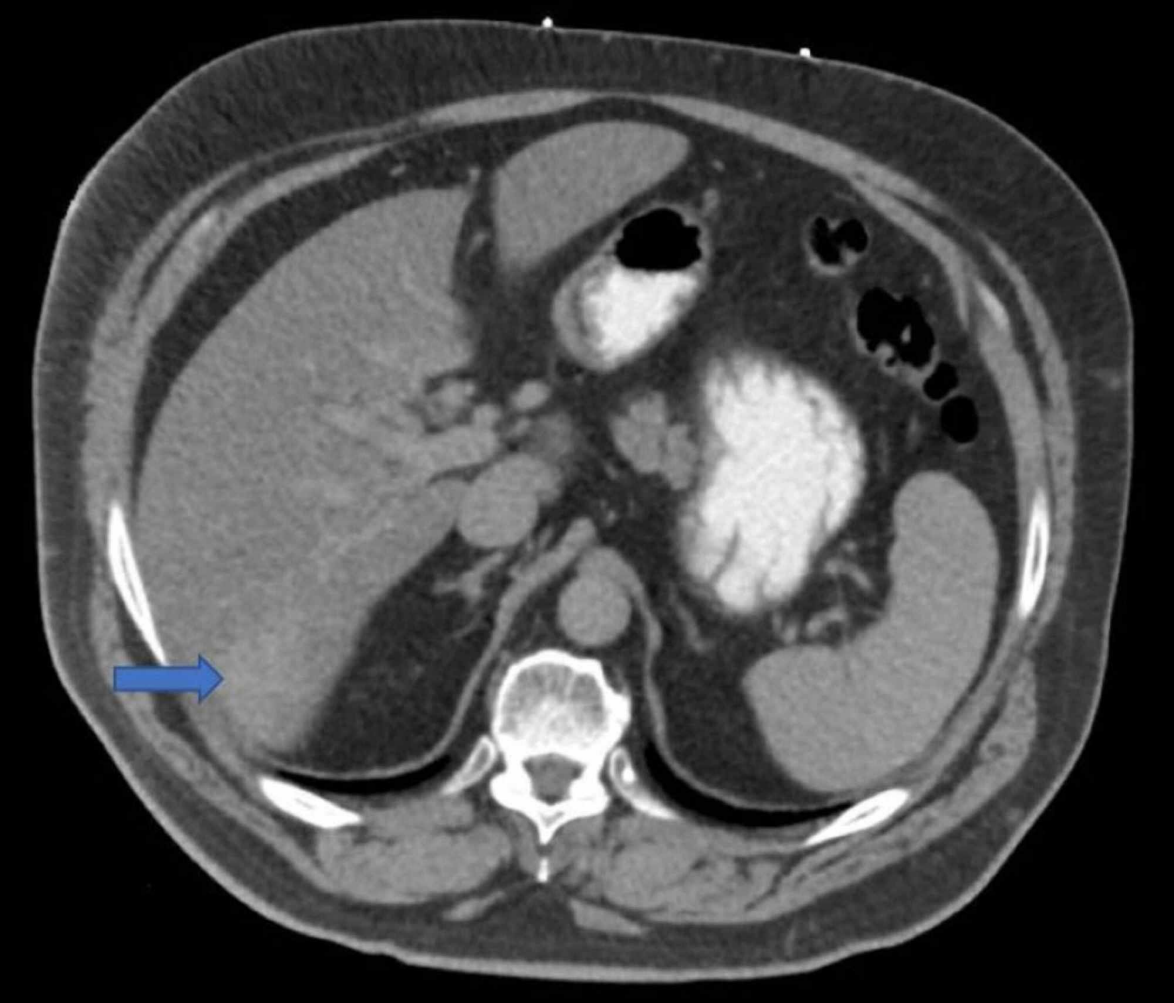
Subtle area of enhancement in the inferior portion of the right hepatic lobe.

A CT scan of the abdomen and pelvis demonstrated subtle areas of enhancement in the right hepatic lobe (Figure 2).

**Figure 3:**
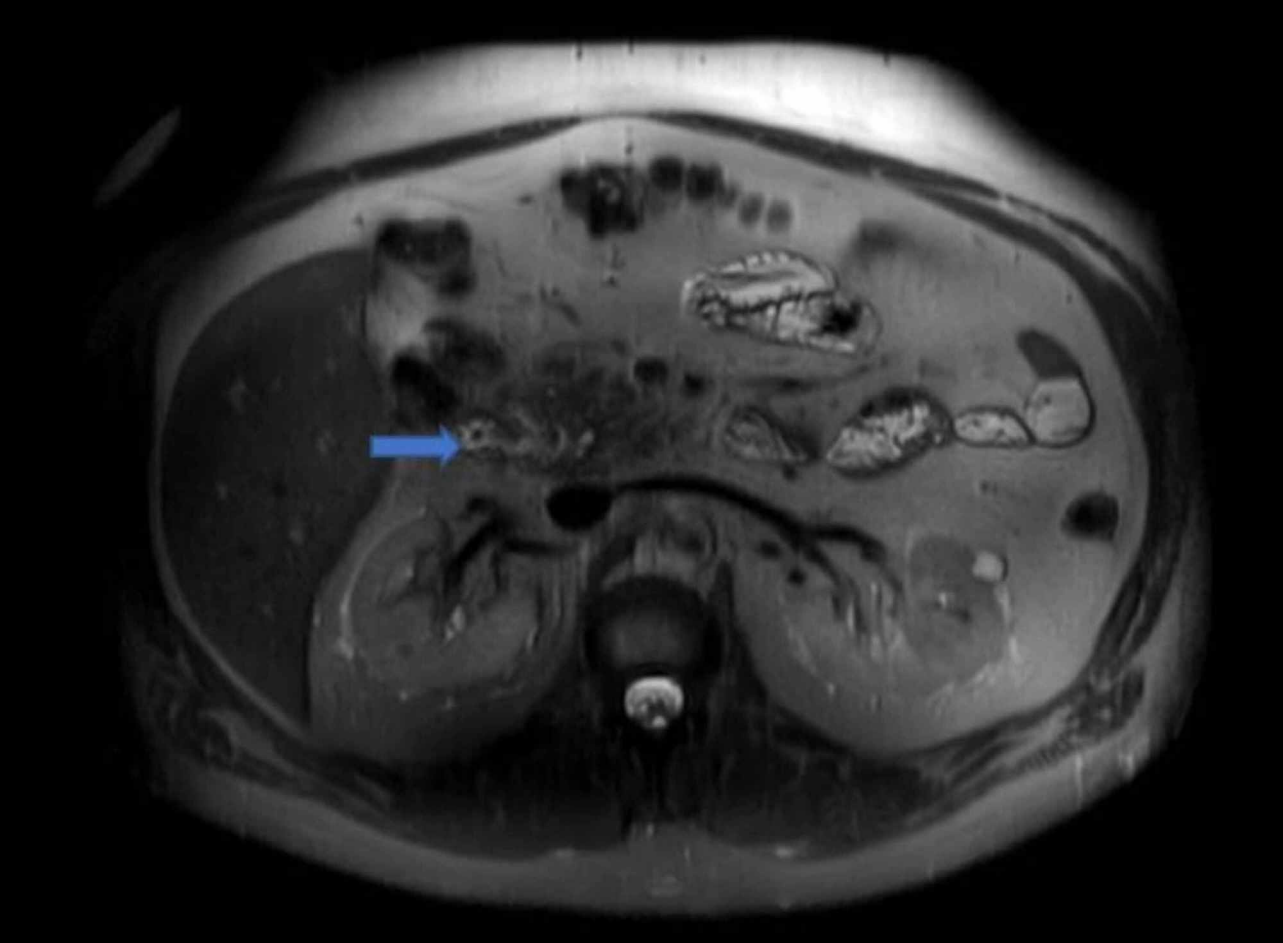
Descending duodenal diverticulum measuring 2 cm.

MRI demonstrated a 15.7-mm hemangioma in the liver with associated hepatomegaly. There was a subtle descending duodenal diverticulum measured to be 2 cm with an associated mild mass effect on the distal ducts (Figure 3).

No choledocholithiasis or focal strictures were seen. The patient was subsequently started on ampicillin-sulbactam. Further workup was unremarkable and included hepatitis panel, antinuclear antibodies, antimitochondrial antibodies, ceruloplasmin, tissue transglutaminase antibody, thyroid-stimulating hormone, and iron profile. ERCP demonstrated a large diverticulum at the second portion of the duodenum with impacted food, also classified as a bezoar, which was removed with rat teeth snare. Mild stenosis at the lower one-third of the main bile duct was tested with a brushing technique for cytology. A plastic stent, with a single external flap and single internal flap, was placed 9 cm into the common bile duct. Bile was noted to flow freely through the stent. Throughout the rest of the patient’s hospitalization, liver enzymes trended down. He was discharged home with a planned follow-up.

## Discussion

PAD denote true extraluminal diverticula of the duodenal mucosa that arise within a 2-3 cm radius from the ampulla of Vater. The majority of cases are asymptomatic. However, approximately 5% of cases have resulted in complications. Cases can be classified as pancreaticobiliary or non-pancreaticobiliary. Pancreaticobiliary complications of PAD center around the obstructive capabilities of the diverticulum, and can manifest as obstructive jaundice, cholangitis, or pancreatitis [1,3]. Non-pancreaticobiliary complications include hemorrhage, fistula formation, perforation, or enterolith formation, which occur secondary to inflammation [3].

Lemmel’s syndrome is specifically classified as obstructive jaundice in the absence of choledocholithiasis or pancreaticobiliary/periampullary tumors. The most likely explanation of Lemmel’s syndrome would be the presence of a PAD, specifically leading to obstruction of the ampulla of Vater [2]. The pathophysiology of Lemmel’s syndrome is dependent on the location of the PAD. In one situation, chronic fibrosis of the papilla might occur secondary to periampullary diverticulitis and chronic inflammation of the ampulla [6]. Additionally, the location of the PAD may cause the sphincter of Oddi to malfunction, leading to a functional obstruction. Alternatively, obstructive jaundice can be the result of external compression of the common bile duct or the ampulla of Vater by a PAD filled with either inflammatory debris, such as pus, or enteroliths [7].

Patients with Lemmel’s syndrome secondary to PAD usually present with right upper quadrant discomfort and jaundice. However, there have been some reports documenting patients who present with painless jaundice [8]. In most cases, laboratory workup reveals leukocytosis, elevated inflammatory markers, such as erythrocyte sedimentation rate and C-reactive protein, elevated direct and total bilirubin, elevated liver enzymes, elevated alkaline phosphatase, and elevated gamma-glutamyl transferase. Elevated pancreatic enzyme levels could be seen with compression of the ampulla of Vater by a PAD [9].

A side-viewing endoscope used during ERCP can help demonstrate the presence of a PAD and is considered the gold standard for diagnosis [2]. Other modalities of imaging, such as a CT scan and magnetic resonance cholangiopancreatography (MRCP), demonstrated that PAD appear as thin-walled cavitary lesions off the medial aspect of the second part of the duodenum [10]. Some PAD can also be filled with fluid, and may resemble pseudocysts, abscesses, or neoplasms. A careful review is necessary to determine whether hyperintensities seen on CT/MRCP are enteroliths encased in a PAD, or otherwise [1].

Treatment and management of patients vary based on symptomology and the pathophysiology of the subtype of Lemmel’s syndrome. In asymptomatic patients, conservative management is recommended. In majority of patients presenting with symptoms indicative of biliary obstruction or cholangitis, endoscopic extraction, extracorporeal shockwave lithotripsy, or surgery, such as a diverticulectomy, may be indicated [1,5,11]. If Lemmel’s syndrome is due to chronic papillary fibrosis or dysfunction of the sphincter of Oddi, then an endoscopic sphincterotomy is preferred [5,11]. 

## Conclusions

Diagnosing Lemmel’s syndrome can be extremely difficult. Cholelithiasis is usually presumed to be the cause of obstructive jaundice. The identification of Lemmel’s syndrome is important in order to avoid delayed management.
